# Quantification of Idua Enzymatic Activity Combined with Observation of Phenotypic Change in Zebrafish Embryos Provide a Preliminary Assessment of Mutated *idua* Correlated with Mucopolysaccharidosis Type I

**DOI:** 10.3390/jpm12081199

**Published:** 2022-07-23

**Authors:** Cheng-Yung Lin, Hsiang-Yu Lin, Chih-Kuang Chuang, Po-Hsiang Zhang, Yuan-Rong Tu, Shuan-Pei Lin, Huai-Jen Tsai

**Affiliations:** 1 Institute of Biomedical Sciences, MacKay Medical College, New Taipei City 25245, Taiwan; tonylin0212@gmail.com (C.-Y.L.); lxc46199@ms37.hinet.net (H.-Y.L.); 2 Department of Medicine, MacKay Medical College, New Taipei City 25245, Taiwan; 3 Department of Pediatrics, MacKay Memorial Hospital, Taipei 10449, Taiwan; 4 Department of Medical Research, MacKay Memorial Hospital, New Taipei City 25160, Taiwan; mmhcck@gmail.com (C.-K.C.); wzmf173814@gmail.com (P.-H.Z.); likemaruko@hotmail.com (Y.-R.T.); 5 MacKay Junior College of Medicine, Nursing and Management, Taipei 11260, Taiwan; 6 Department of Medical Research, China Medical University Hospital, China Medical University, Taichung 40402, Taiwan; 7 School of Medicine, Fu-Jen Catholic University, New Taipei City 242062, Taiwan; 8 Department of Infant and Child Care, National Taipei University of Nursing and Health Sciences, Taipei 11219, Taiwan; 9 Department of Life Science, Fu Jen Catholic University, New Taipei City 242062, Taiwan

**Keywords:** enzymatic activity, IDUA, mucopolysaccharidosis type I, overexpression, zebrafish

## Abstract

Mucopolysaccharidosis type I (MPS I) is an inherited autosomal recessive disease resulting from mutation of the α-l-Iduronidase (IDUA) gene. New unknown mutated nucleotides of *idua* have increasingly been discovered in newborn screening, and remain to be elucidated. In this study, we found that the z-Idua enzymatic activity of zebrafish *idua*-knockdown embryos was reduced, resulting in the accumulation of undegradable metabolite of heparin sulfate, as well as increased mortality and defective phenotypes similar to some symptoms of human MPS I. After microinjecting mutated z-*idua*-L346R, -T364M, -E398-deleted, and -E540-frameshifted mRNAs, corresponding to mutated human *IDUA* associated with MPS I, into zebrafish embryos, no increase in z-Idua enzymatic activity, except of z-*idua*-E540-frameshift-injected embryos, was noted compared with endogenous z-Idua of untreated embryos. Defective phenotypes were observed in the z-*idua*-L346R-injected embryos, suggesting that failed enzymatic activity of mutated z-Idua-L346R might have a dominant negative effect on endogenous z-Idua function. However, defective phenotypes were not observed in the z-*idua*-E540-frameshifted-mRNA-injected embryos, which provided partial enzymatic activity. Based on these results, we suggest that the z-Idua enzyme activity assay combined with phenotypic observation of mutated-*idua*-injected zebrafish embryos could serve as an alternative platform for a preliminary assessment of mutated *idua* not yet characterized for their role in MPS I.

## 1. Introduction

Mucopolysaccharidosis type I (MPS I) is a lysosomal storage disorder (LSD) caused by mutations in the *IDUA* gene coding for the enzyme α-l-Iduronidase (IDUA) involved in the degradation of glycosaminoglycans (GAGs), such as heparan sulfate (HS) and dermatan sulfate (DS) in lysosome. Mutations in *IDUA* impair the degradation of HS and DS, resulting in their accumulation within lysosomes, in turn, causing chronic and progressive dysfunction of cells, tissues, and organs [1,2]. MPS I is an inherited autosomal recessive disease, and its clinical spectrum varies from severe Hurler syndrome (MPS I-H; OMIM #67014) to attenuated Scheie syndrome (MPS I-S; OMIM #67016) with an intermediate disease phenotype classified as Hurler–Scheie syndrome (MPS I-HS; OMIM #67015) [3,4]. The phenotypic manifestations of MPS I are upper airway obstruction, laryngeal and tracheal narrowing, hearing and visual deficit, gargoyle facies, organomegaly, abdominal herniae, valve disease and cardiomyopathy, and skeletal deformities such as thoracolumbar gibbus and joint stiffness. MPS I-H also presents severe neurocognitive decline. Death, typically caused by cardiorespiratory failure, usually occurs within the first ten years of life [1,2,5,6].

Currently approved treatments consist of hematopoietic stem cell transplantation (HSCT) and enzyme replacement therapy (ERT). Although HSCT and ERT significantly improve disease manifestations and prolong life, these treatments may only prevent MPS I manifestations from worsening, but it is impossible to recover completely. Owing to nonspecific early symptoms [7,8], mutational screening of MPS I in newborns might be an effective approach for reducing diagnostic delay [9,10,11]. As a result of such a newborn screening program [9,12], at least more than 199 mutations have been identified in the *IDUA* gene, including missense/nonsense mutations, deletions, insertions, and rearrangements [13]. Although several *IDUA* mutations have been confirmed from clinical symptoms [9], most remain to be elucidated for causing MPS I. Thus, apart from the conventional diagnostic approaches, this calls for the development of a fast, but effective, in vivo platform that can preliminarily assess which mutated nucleotides of *IDUA* might be tightly correlated with the occurrence of MPS I.

The lower vertebrate zebrafish possesses many advantages over higher vertebrates, such as low maintenance, high fecundity, light-induced spawning, transparent embryos, short generation interval, rapid embryonic development, fully sequenced genome, and some phenotypes similar to human diseases [14]. Such merits have popularized the zebrafish as a model system for biomedical and pharmaceutical studies, including drug screening [15,16]. Additionally, zebrafish are regarded as an important research tool for studying LSD [17,18]. The MPS zebrafish model has been extensively used to study iduronate 2-sulfatase (IDS)-associated Hunter syndrome (MPS II). For example, the knockdown of zebrafish *ids* (*z-ids*) by injection of an antisense morpholino oligonucleotide (MO) leads to increased mortality and severe developmental defects. These include disruption of body axis organization, abnormal heart morphogenesis, and malformation of craniofacial cartilage in zebrafish larvae, which are mostly phenocopied in MPS II clinical symptoms [19,20,21]. Similar to valve disease that occurs in MPS II, loss of z-Ids function causes the defective atrioventricular valve in zebrafish embryos owing to abnormal early Sonic Hedgehog and Wnt/b-catenin signaling [20]. Bellesso et al. [22] employed CRISPR/Cas9 technology to generate a transgenic zebrafish line producing a truncated form of z-Ids in cells. They demonstrated that loss of z-Ids function in the early developmental stage of zebrafish decreased Fgf signaling, which, in turn, caused scoliosis and kyphosis at the later developmental stages. Furthermore, Lin et al. [23] employed a gain-of-function strategy through microinjection of different mutated *z-ids* mRNAs, corresponding to mutated nucleotides that occur in the human *IDS* gene, into zebrafish embryos. They found that z-Ids enzymatic activity was not increased in embryos overexpressing z-*ids* with a missense mutation. However, it was increased in embryos injected with a nonsense mutation that could maintain z-Ids enzymatic function. More importantly, overexpression of missense mutant z-*ids* caused the phenotypic defects of zebrafish embryos since this null function of mutant z-Ids competes with the endogenous normal z-Ids, impeding the normal function of z-Ids. Therefore, this approach provides an alternative platform for quickly assessing the point mutation of z-*ids* gene that has not yet been characterized.

Recently, Douek et al. [24] generated a *sgsh*^Δex5−6^ zebrafish mutant by CRISPR/Cas9 to study MPS IIIA and found that it exhibited central nervous system (CNS)-specific defective features, including neuronal lysosomal overabundance, complex behavioral phenotypes, and neuroinflammation. They also found that the pharmacological inhibition of Caspase-1 could partially rescue the behavioral abnormality that occurred in *sgsh*^Δex5−6^ mutant larvae.

The overall birth incidence for all patients with MPS was reported to be 2.04 per 100,000 live births in Taiwan [25], which was close to that of MPS in European countries, ranging from 1.75 (Sweden) to 4.5 (The Netherlands) per 100,000 live births [26,27,28]. Of these cases, MPS II, comprising 52% of all diagnosed MPS cases, has the highest birth incidence (1.07 per 100,000 live births). However, the birth incidence rate of MPS I was 0.11 per 100,000 live births, accounting for 6% of all MPS cases [21,25]. These data were close to those reported in Japan and South Korea [29], but with a significant difference from those of European and American countries [9,30]. Unlike extensive studies reporting on MPS II, as described in the previous section, using a zebrafish in vivo model to study z-Idua function in MPS I is, to the best of our knowledge, absent. Therefore, in this study, we employed a loss-of-function strategy in zebrafish embryos to detect z-Idua enzymatic activity and observe the defective phenotypes. Additionally, we used the gain-of-function approach to build an in vivo assay platform to determine the effect of different mutated z-*idua* nucleotides on z-Idua enzymatic activity and phenotypic change to provide a valuable index for determining z-*idua* mutations that might be essential to the occurrence of MPS I in humans.

## 2. Materials and Methods

### 2.1. Ethics Statement

The MacKay Memorial Hospital Institutional Animal Care and Use Committee (IACUC) reviewed and approved the protocol described below (MMH-A-S-111-38), approved on 31 May 2022.

### 2.2. Fish Embryos

The wild type zebrafish AB strain were purchased from University of Oregon, USA. The culture condition, embryo stage, egg production and collection were following the description reported by Westerfield [31]. The morphological phenotypes of embryos were observed under a fluorescent stereomicroscope (Leica MZ FLIII, Berlin, Germany).

### 2.3. Knockdown of z-idua in Zebrafish Embryos 

The antisense morpholino oligonucleotide (MO) 5′-GAACGGAGTTTACATTTGCACATGC-3′ used to specifically knock down translation of zebrafish *idua* mRNA (z-*idua*) was synthesized by Gene-Tools Inc. (Philomath, OR, USA) and prepared at a stock concentration of 1 mM and diluted to 8, 12, or 16 ng/uL. Each microinjection into one- to two-cell-stage zebrafish embryo was 2.3 nL.

### 2.4. LC-MS/MS Assay and Calibration of DS and HS

Experimental parameters of LC-MS/MS assay basically followed the report published previously by Chuang et al. [32] with some modifications specific for zebrafish embryos’ extracts. LC-MS/MS analysis was performed on an AB 4000 QTRAP LC-MS/MS System (AB Sciex, Framingham, MA, USA) equipped with a TurboIonSpray (electrospray ionization; ESI), and Agilent 1260 Infinity HPLC pump and autosampler (Agilent Technologies, Santa Clara, CA, USA). An Atlantis dC18 3 μm column (3.0 × 50 mm; Waters Corporation, Milford, MA, USA) was used for DS and HS analysis. Data were acquired and processed using Analyst 1.5.2 TM software (AB Sciex, Framingham, MA, USA). Each batch analysis of DS and HS standards was calibrated with five known concentrations mixed working standards of 12.5, 25, 50, 100, and 200 μg/mL and 6.25, 12.5, 25, 50, and 100 μg/mL for DS and HS, respectively. Total proteins prepared for each reaction were extracted from 40 zebrafish embryos at 120 hpf.

### 2.5. RNA Extraction and Reverse Transcriptase Polymerase Chain Reaction (RT-PCR)

Total RNA isolation from embryos, cDNA synthesis, and RT-PCR were performed as previously described [33]. The primer set used for RT-PCR for molecular cloning of z-*idua* cDNA (XM_001923654.7) fused with reporter FLAG peptide was: forward primer (5′-TATATATCGATATGTGTAAGTGCAAGTTAAGATCATTGACATGG-3′) and reverse primer (5′-TATATCTCGAGGGAAAGTCAAAAGCCTAACTGTGACTACTTATCGTCGTCATCCTTGTAATCC-3′).

### 2.6. Plasmid Constructs Containing idua cDNA and Its Mutated Nucleotides

The cDNA of zebrafish *idua* fused with reporter FLAG peptide was amplified by PCR using primers listed in Section 2.5 under the following condition: initial denaturation at 98 °C for 5 min; 30 cycles of denaturation at 98 °C for 1 min, annealing at 60 °C for 1 min, and elongation at 72 °C for 2 min; with a final cycle of extension at 72 °C for 5 min. The PCR product with 3753 bp molecular size was restricted by *Cla*I/*Xho*I and engineered into the corresponding cutting sites of plasmid pCS2^+^ vector to generate plasmid pCS2-z-*idua*. Four mutated forms of z-*idua* (L346R, T364M, E398 deletion, and E540 frameshift), corresponding to the mutated sites of human *IDUA* causing MPS I, were also obtained by PCR using primers listed in Table 1 under the condition described above. The mutagenesis to z-*idua* was conducted using PCRs and annealed two newly synthesized PCR products. The PCR product was cut with *Cla*I/*Xho*I and subcloned into the corresponding cut of pCS2^+^ vector to generate plasmids of pCS2-z-*idua*-L346R, -T364M, -E398-del and E540-fra.

### 2.7. Overexpression of z-Idua mRNA

Capped mRNAs encoding wild type z-Idua and their mutated or truncated forms were synthesized according to the protocol provided by the manufacturer (Epicentre). The resultant z-*idua* mRNA and its derivates were diluted to 22, 44, or 88 ng/µL with double distilled water and microinjected into a one- to two-cell-stage embryo in a volume of 2.3 nl per embryo. 

### 2.8. Enzyme Activity of z-Idua Contained in Zebrafish Embryos

The method was basically following the report published by Chamoles et al. [34] and Lin et al. [35] with some modifications for measuring the total proteins extracted from zebrafish embryos. The zebrafish embryos at 24 h post-fertilization (hpf) were lysed with whole-cell extraction buffer (20 mM HEPES, 0.2 M NaCl, 0.5% Triton X-100, 20% glycerol, and 1 mM EDTA). The substrate of the IDUA, 4-methylumbelliferyl alpha-L-iduronide (4MU-I, Toronto Research Chemicals, ON, Canada, #M334701), was diluted with sodium formate buffer (50 mM, pH 2.8) to the final volume of 20 μg/20 μL. The 20 μL aliquots of 4MU-I were mixed with 20 μg/20 μL sample of tissue homogenates prepared by Microtube Pellet Pestle Rods with Motor (Sansho, Osaka, Japan). The mixture was incubated at 37 °C for 20 h, and then 200 μL glycine carbonate buffer (pH 10.5) was added to terminate the reaction. The intensity of emitted fluorescence resulting from the enzyme-substrate reaction was measured with excitation at 365 nm and emission at 450 nm. A standard curve was formulated by 4MU (Sigma-Aldrich, St. Louis, MO, USA, #M1381). Z-Idua enzyme activity was expressed as units per mg proteins extracted from zebrafish embryos after reaction for 20 h. The calculation of relative enzyme activity of untreated WT control embryos was normalized as 1 for comparison with the relative z-Idua activity obtained from the other experimental groups.

### 2.9. Cartilage Staining

Alcian blue staining was conducted following the protocol described by Lin et al. [36] with some modifications. Embryos at 120 hpf were anesthetized using 0.02% buffered tricaine (Sigma) and fixed overnight in 4% paraformaldehyde (PFA) at 4 °C. After washing with phosphate-buffered saline (137 mMNaCl, 2.7 mMKCl, 10 mM Na_2_HPO_4_·2H_2_O, 2 mM KH_2_PO_4_), embryos were stained overnight in 0.1% alcian blue, which was dissolved in acidic ethanol (70% ethanol, 5% concentrated hydrochloric acid), then washed extensively in acidic ethanol, dehydrated, digested with 0.02% trypsin, and stored in 80% glycerol. 

### 2.10. Histological Examination, Frozen Section, and Immunostaining of Zebrafish Embryos

For the histological examination of eye development in z-*idua*-injected zebrafish embryos, the samples were fixed in 4% PFA at 4 °C for 24 h, embedded in paraffin, cut into sections 40-μm thick, and then stained with Hematoxylin and Eosin. The procedures of frozen dissection and immunostaining were previously described by Lin et al. [37] except that samples were cut into coronal sections 30 μm-thick, followed by immunofluorescence staining. For the detection of rod photoreceptors, the primary antibody against zebrafish Zpr3 (ZIRC, ZDB-ATB081002-45, 1:100) and secondary antibody against mouse Cy3 (Sigma, AP124C, 1:100) were used. For the detection of cell nuclei, DAPI was used to stain for 20 min. The patterns were observed under a laser scanning confocal fluorescence microscope (Zeiss, Oberkochen, Germany).

## 3. Results

### 3.1. The Dosage of z-idua mRNA Injected into Embryos Was Positively Correlated with the Enzymatic Activity of IDUA Detected in Zebrafish Embryos

**Figure 1:**
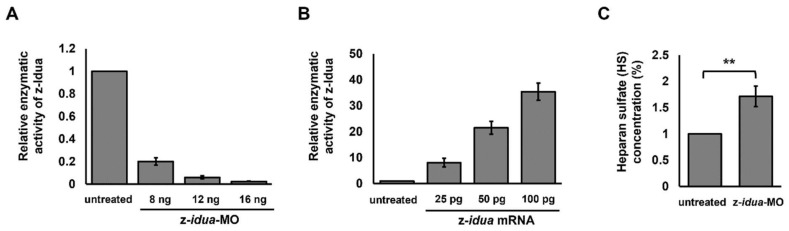
Both z-Idua enzymatic activity and the accumulation of heparan sulfate (HS) in zebrafish embryos were closely correlated with knockdown or overexpression of z-*idua* mRNA injected into zebrafish embryos. (**A**) Zebrafish embryos at one-cell stage were injected with different doses of z-*idua*-MO, as indicated, followed by detection of z-Idua enzymatic activity at 24 hpf. The z-Idua enzymatic activity of the untreated control group was normalized as 1 for comparison with embryos injected with different doses of z-*idua*-MO. (**B**) The z-Idua enzymatic activity of zebrafish embryos injected with different doses of z-*idua* mRNA, as indicated. The z-Idua enzymatic activity of the untreated control group was normalized as 1 for comparison with the embryos injected with different doses of z-*idua* mRNA. (**C**) HS concentration was detected at 120 hpf of zebrafish embryos injected with 12 ng of z-*idua*-MO. The HS concentration of the untreated control group was normalized as 1 for comparison with the z-*idua*-MO-injected embryos. One trial experiment was carried out for 40 larvae. Data of each group were averaged from three independent experiments. Student’s *t*-test was used to determine significant differences between the two groups (**, *p* < 0.01).

The amino acid sequence of zebrafish Idua (z-Idua) shared 70% similarity with that of human IDUA (h-IDUA). Bie et al. [38] predicted that the enzyme catalytic sites of h-IDUA were at Glu 182 and Glu 299 and that the N-glycosylation site, which affects enzyme catalytic activity [39], was at Asn 372, whereas the corresponding sites for z-Idua were Glu 182, Glu 299, and Asn 372 (XM_021474008), respectively, suggesting they were highly conserved (Supplemental Figure S1). To study z-Idua function, we first detected the enzymatic activities of z-Idua in WT zebrafish embryos and embryos injected with z-*idua*-morpholino (MO) at the dosage of 8, 12, and 16 ng per embryo. Total proteins of embryos were extracted at 24 hpf, and their enzymatic activities were quantified (Figure 1A). When z-Idua enzymatic activity in the WT control group was normalized to 1, it was reduced to 0.23, 0.05, and 0.01 in embryos injected with 8, 12, and 16 ng z-*idua*-MO, respectively (Figure 1A). This line of evidence suggested that the degree of reduced enzymatic activity of z-Idua in zebrafish embryos was dependent on the amount of z-*idua*-MO injected into embryos.

Next, we overexpressed z-*idua* in embryos by injecting z-*idua* mRNA into one-cell stage zebrafish embryos. When z-Idua enzymatic activity in the WT control group at 24 hpf was normalized to 1, the relative z-Idua enzymatic activity of embryos injected with 25- to 100-pg z-*idua* mRNA was increased by 8.1 ± 1.7- to 35.5 ± 3.3-fold, respectively (Figure 1B). These data suggested that the increase of z-Idua enzymatic activity in zebrafish embryos was also dependent on the amount of z-*idua* mRNA injected. 

Furthermore, since the accumulation of DS and HS occurred in the blood and urine of MPS I patients, we used liquid chromatography/tandem mass spectrometry (LC-MS/MS) to detect the concentration of DS and HS in z-*idua*-MO-injected zebrafish embryos at 120 hpf. Compared with the WT control group, results showed that the concentration of HS in the total proteins of z-*idua*-MO-injected embryos increased 1.7 ± 0.2-fold (Figure 1C), suggesting that the lack of z-Idua resulted in the accumulation of HS. However, the concentration of DS in z-*idua*-MO-injected embryos was not significantly different from that of WT.

### 3.2. Using Zebrafish Embryos Served as an In Vivo Platform to Analyze the Enzymatic Activity of Mutant z-Idua

**Figure 2:**
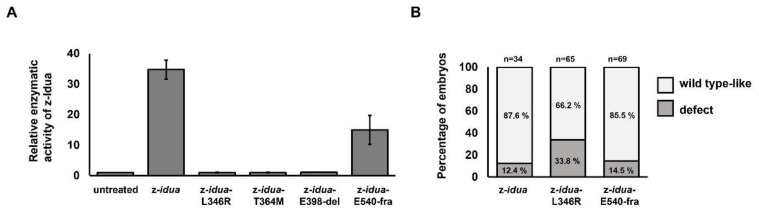
The z-Idua enzymatic activity and occurrence of defective phenotypes in zebrafish embryos was impacted by injection of mutated z-*idua* mRNA into embryos. (**A**) The z-Idua enzymatic activity of untreated zebrafish embryos at 24 hpf, which served as the control group, was quantified and normalized as 1 for comparison with the relative z-Idua activity obtained from the other experimental groups. The z-Idua enzymatic activity of embryos injected with 100-pg zebrafish *idua* mRNA (z-*idua*; served as positive control) and various point-mutated z-*idua* mRNAs, as indicated, were quantified. Each point-mutation that occurred in z-*idua* corresponded with that of human MPS I patients. (**B**) Embryonic phenotypes were examined at the 120 hpf zebrafish embryos injected at one-cell stage with 100-pg zebrafish *idua* mRNAs (z-*idua*), including mutated variants, as indicated. Each mutated zebrafish *idua*.

Thus far, our results show that either overexpression or knockdown of z-*idua* mRNA in zebrafish embryos positively correlates with their enzymatic activity. Therefore, we went further to determine whether zebrafish embryos could serve as an in vivo platform to evaluate the effects of the mutated *idua* gene on enzymatic activity. To accomplish this, we designed mutant zebrafish z-*idua* mRNA, such as z-*idua*-L346R (human reference SNP number: rs121965033), z-*idua*-T364M (human reference SNP number: rs121965032), z-*idua*-E398-del (deletion) (human *IDUA* c.1192_1194delGAG; p.E398del), and z-*idua*-E540-fra (frameshift) (human *IDUA* c.1634delA c.1634_35insGGG; p.E545Gfs*16), corresponding to mutant sites that cause human MPS I [40,41,42]. These mRNAs were injected into zebrafish embryos, and their z-Idua enzymatic activities were detected at 24 hpf. When the z-Idua enzymatic activity of untreated WT control embryos was normalized as 1, results showed that the z-Idua enzymatic activity of embryos injected with normal z-*idua* mRNA increased to 34.73 ± 3.1 (Figure 2A). However, the relative z-Idua enzymatic activities in the z-*idua*-L346R, z-*idua*-T364M, z-*idua*-E398-del, and z-*idua*-E540-fra embryos were 0.99 ± 0.1, 1.01 ± 0.1, 1.15 ± 0.1, and 15.00 ± 4.8, respectively (Figure 2A). This line of evidence demonstrated that overexpression of z-*idua* mRNA increased the enzymatic activity of z-Idua, whereas overexpression of z-*idua*-L346R, z-*idua*-T364M, and z-*idua*-E398-del did not change z-Idua enzyme activity, suggesting that these three mutated sites could directly cause the loss of z-Idua enzymatic activity. However, we noticed that z-Idua enzymatic activity was partially retained in the z-*idua*-E540-fra-overexpressed embryos.

### 3.3. Effect of Overexpression of Two Zebrafish Mutated idua mRNAs, Corresponding to Human Mutated IDUA Genes, on the Morphological Change in Zebrafish Embryos

To study the effect of overexpression of mutated *idua* on the morphological change of zebrafish embryos, we microinjected two mutated z-*idua* mRNAs, including z-*idua*-L346R and -E540-fra, corresponding to h-*idua*-L346R and h-*idua*-E545-fra, respectively, reported as MPS I homologous pathogenic sites. Results showed that the percentage of defective embryos observed in total embryos injected with normal z-*idua* mRNA embryo was 12.4% (*n* = 34) (Figure 2B). Compared with embryos injected with normal z-*idua*, the percentage of the defective phenotype observed in all embryos injected with the two mutated z-*idua* mRNAs was 33.8% (*n* = 65) and 14.5% (*n* = 69) for z-*idua*-L346R and -E540-fra, respectively (Figure 2B), suggesting that overexpression of mutated z-Idua-L346R protein led to a significant increase in the occurrence of embryo abnormality during development.

### 3.4. Knockdown of z-Idua in Zebrafish Embryos Caused Developmental Defects

**Figure 3:**
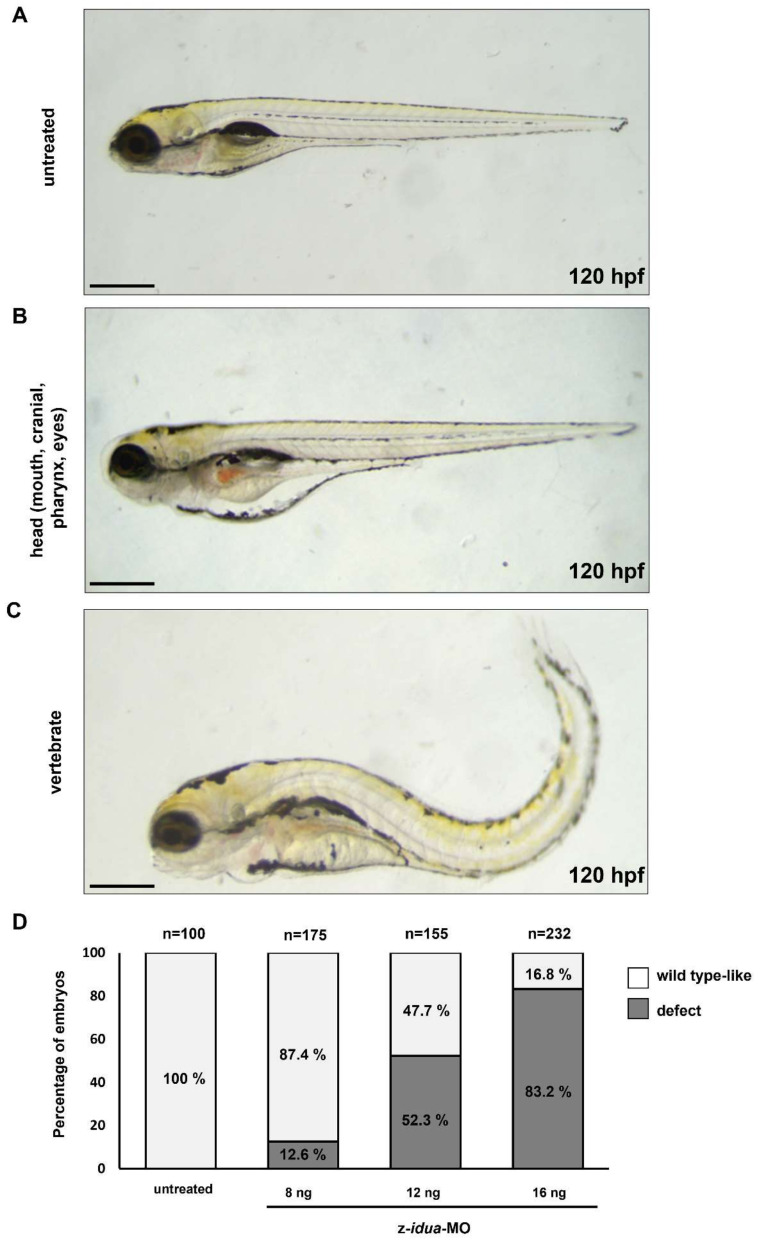
Knockdown of z-*idua* caused defective development in zebrafish embryos. Embryonic phenotypes were observed in 120 hpf zebrafish embryos injected with 12 ng z-*idua*-MO. (**A**) Untreated control embryos; (**B**,**C**): z-*idua*-MO-injected embryos; (**B**) defective head, including smaller eyes, abnormal craniofacial and pharyngeal arches, and cardiac edema; (**C**) defective body shape, including shortened somites and bent body axis. (**D**) Percentage of defective phenotypes occurring in all examined embryos injected with z-*idua*-MO. Embryonic morphology was examined at 120 hpf in the zebrafish embryos injected at one-cell stage with different concentrations of z-*idua*-MO, as indicated. The occurrence rates (in percentages) of defective phenotypes (marked in black boxes) and the wild type-like phenotype (marked in grey) among the total examined embryos injected were calculated. The total number (*n*) of embryos studied in each group was indicated at the top of each column. Scale bar: 500 μm.

To understand whether the absence of z-Idua would lead to the occurrence of defective phenotypes in zebrafish embryos, we injected z-*idua*-MO into one-cell stage zebrafish embryos to inhibit the function of z-Idua and observed the detective phenotypes at 120 hpf. Compared with the untreated WT embryos (Figure 3A), we found two major phenotypes in the z-*idua*-MO-injected embryos: (1) defective head, including reduced craniofacial size, abnormal pharyngeal arches, smaller eyes and edema (Figure 3B), and (2) defective vertebrates, i.e., bent body axis (Figure 3C). Lack of z-Idua function caused serious defects in embryos. Increased concentration of injected z-*idua*-MO from 8 to 16 ng led to an increase in defect percentage among injected embryos from 12.6% (*n* = 175) to 83.2% (*n* = 232). The occurrence of developmental defects was dose-dependent and suggested that the observed phenotypes could be attributed to the absence of z-Idua in embryos.

**Figure 4:**
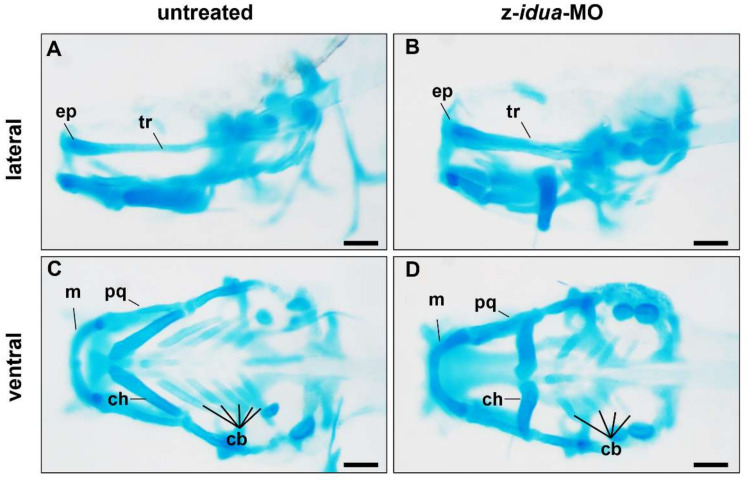
Knockdown of z-*idua* caused defective head cartilage in zebrafish embryos. Using alcian blue staining to detect the head cartilage of zebrafish embryos at 120 hpf. Untreated embryos (control) and embryos injected with antisense morpholino oligonucleotides (MO) complementary with z-*idua* (z-*idua*-MO) were studied. (**A**,**B**) Lateral view; (**C**,**D**) Ventral view; (**A**,**C**) Untreated control embryos; and (**B**,**D**) z-*idua*-MO injected embryos. Loss of function of z-Idua caused deformed cranial cartilage and decreased number of cartilage cells. cb, ceratobranchial; ch, ceratohyal; ep, ethmoid plate; m, Meckel’s cartilage; pq, palatoquadrate; tr, trabecular plate. Scale bar: 100 μm.

Since the craniofacial defect was observed in the z-*idua*-knockdown embryos, we employed alcian blue stain to study the head cartilage of zebrafish embryos. Compared with 120 hpf untreated WT embryos (*n* = 5) (Figure 4A,C), the bone length of trabecular plate (tr), which support forehead and eye socket in the z-*idua*-MO-injected embryos, was decreased 8% (*n* = 5) (Figure 4B,D). In the lower jaw region, the bone length of Meckel (m) and palatoquadrate (pq) was reduced by 3 and 20%, respectively (*n* = 5) (Figure 4B,D). In the pharyngeal arch region, the pattern and distribution of ceratohyal (ch) cartilage was altered, and the number of ceratobranchials (cb) was reduced to 32% (*n* = 5) (Figure 4B,D). These data suggested that the knockdown of z-*idua* impaired the number and morphology of craniofacial cartilage in zebrafish embryos.

**Figure 5:**
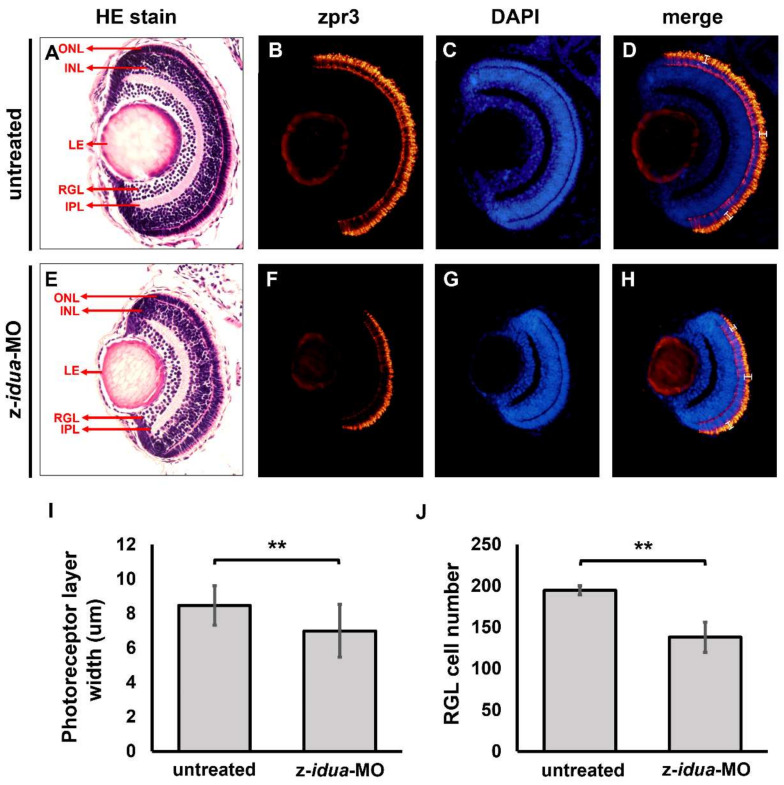
Knockdown of z-*idua* caused defective eyes in zebrafish embryos. Using hematoxylin and eosin staining (HE stain) and immunofluorescent staining to examine the ocular morphology of zebrafish embryos at 120 hpf. (**A**–**D**) untreated control groups; (**E**–**H**) z-*idua*-knockdown embryos. (**A**,**E**) HE staining; (**B**,**F**) immunofluorescent stain using zpr3 antibody to detect rod cells labeled in red fluorescence; (**C**,**G**) DAPI-marked nucleus labeled in blue fluorescence; (**D**,**H**) two signals merged. (**I**) To quantify the width of photoreceptor layer. (**J**) To quantify the cell number in retinal ganglion layer (RGL). All data were averaged from five independent experiments and represented as mean ± S.D. Student’s *t*-test was used to determine significant differences between each group (**, *p* < 0.01). ONL, outer nuclear layer; INL, inner nuclear layer; LE, lens; RGL, retinal ganglion layer; IPL, inner plexiform layer.

Lastly, we performed transection and histological staining to examine in more detail the structure of eyes from z-*idua*-MO-injected embryos. Compared with 120 hpf untreated WT embryos (*n* = 5) (Figure 5A–D), we found that the eyes of z-*idua*-MO-injected embryos were smaller in appearance, but each ocular layer within the eyes was distinctly formed (Figure 5E–H). However, the thickness of the photoreceptor layer in the z-*idua*-MO-injected embryos was reduced from 8.5 ± 1.15 μm (*n* = 5) (Figure 5D,I) to 7 ± 1.53 μm (*n* = 5) (Figure 5H,I). Additionally, the cell number of the retinal ganglion layer in the z-*idua*-MO-injected embryos was reduced from 194.5 ± 5.6 (*n* = 5) (Figure 5D,J) to 138 ± 18.2 (*n* = 5) (Figure 5H,J). In summary, we concluded that the loss of function of z-Idua in zebrafish embryos caused the reduction in (1) eye size, (2) thickness of the photoreceptor layer, and (3) cell number of retinal ganglion layer

## 4. Discussion

Mutational screening for MPS I in newborns is an effective approach for reducing diagnostic delay and preventing phenotypic manifestations from worsening. Hundreds of mutations identified in *IDUA* from newborn screening are necessary to be characterized. Here, for the first time, we used a gain-of-function approach to build an in vivo assay platform to determine the effect of different mutated z-*idua* nucleotides on z-Idua enzymatic activity and phenotypic change in zebrafish embryos to provide a valuable index for determining z-*idua* mutations that might refer to the occurrence of MPS I in humans.

### 4.1. Molecular Strategies Used to Determine Gene Function in Zebrafish Embryos Could Also Be Employed to Identify Gene Function of Mutated Human IDUA

Human IDUA and zebrafish Idua not only share high similar amino acid sequences, including enzymatic catalytic sites at Glu 182 and Glu 299 and an N-glycosylation site at Asn 372 (Supplemental Figure S1), but also have similar enzymatic functions. Thus, the conventional methodologies applied for quantifying enzymatic activity of human IDUA could also be employed to quantify the enzymatic activity of zebrafish Idua (Figure 1). This means that both gain- and loss-of-function strategies used to determine gene function in zebrafish embryos could also be employed to identify the gene function of mutated *idua* gene of humans. For example, knockdown of z-*idua* by injection of MO reduces z-Idua enzymatic activity, whereas overexpression of z-*idua* by injection of mRNA increases it. Using genetic engineering, mutated z-*idua*, corresponding to mutated h-*IDUA*, could be generated and microinjected into zebrafish embryos, followed by enzymatic assay and phenotypic observation.

### 4.2. Endogenous z-Idua Enzymatic Activity Did Not Increase in Embryos Injected with Mutant mRNAs Having Null Enzymatic Function

In this study, we overexpressed four mutated z-*idua* mRNAs, corresponding to four mutated *h-IDUA* mRNAs. Two mutations, L346R (rs121965033) and T364M (rs121965032), were well documented previously [41,42], while the other two mutations, E398-del and E540-fra, newly discovered in 2019 [40], were chosen from a databank. Compared with untreated control zebrafish embryos, results showed no additive effect on endogenous z-Idua enzymatic activity in embryos microinjected with mRNAs from z-*idua*-L346R, z-*idua*-T364M, and z-*idua*-E398-del. However, additive z-Idua enzymatic activity was exhibited in the z-*idua*-E540-fra-mRNA-injected embryos, although not reaching the level of embryos injected with normal z-*idua* (Figure 2). Therefore, it can be concluded that mutants z-*idua*-L346R, z-*idua*-T364M, and z-*idua*-E398-del completely lost the enzymatic activity of Idua. We speculate that the alternation of essential amino acids at the Triosephosphate Isomrase Barrel domain ranging from 42 to 396 amino acid residues of IDUA [39], a major enzymatic activity region, results in loss of enzymatic function, such as h-IDUA-L346R and -T364M proteins. It is also known that h-IDUA-L346R mutation might cause the steric hindrance of h-IDUA and destabilize the enzymatic active site conformation [13,38]. In contrast, the IDUA translated from mutant z-*idua*-E540-fra mRNA retained partial function. This partial retention was responsible for increasing endogenous z-Idua enzymatic activity, as shown in Figure 1. We speculate that the frameshift at E540 of z-Idua protein could still possess partial functionality because this frameshifted form of IDUA only loses the type III fibronectin-like domain ranging from 546 to 642 amino acid residues [39] without altering the major enzymatic activity domain of IDUA, and in result could partially keep its enzymatic function.

### 4.3. The Occurrence of Morphological Defect Was Correlated with the Overexpression of Mutant z-Idua Having Null Enzymatic Function in Embryos

Functional screening for mutated *idua* genes is another advantage of this zebrafish in vivo platform because it can be used to determine if morphological defects are induced by the overexpression of mutated z-*idua* within five days. For example, the injection of z-*idua* mRNA (100 pg/embryo) caused no morphological changes during the development of zebrafish embryos, suggesting that overexpressed normal z-Idua protein in the embryo does not interfere with embryonic development. On the other hand, whereas overexpressed mutant z-Idua-L346R protein resulted in a higher proportion of defective phenotypes of embryos, overexpressed mutant z-Idua-E540-fra protein did not (Figure 2). We interpret these results to mean that increased null enzymatic activity of mutant z-Idua-L346R in embryos might compete with the endogenous normal function of z-Idua, leading to failed hydrolyzation of DS and HS or blockage of z-Idua involved in normal development. In contrast, increased partial enzymatic activity of mutant z-Idua-E540-fra protein in embryos might partially contribute to DS and HS hydrolyzation, leading to normal embryonic development with no signs of phenotypic change. 

Interestingly, the IDUA mutated at L346R was found in patients with a severe form of Hurler syndrome [41], whereas the IDUA-E540-fra mutant was found in patients with a milder form of Hurler syndrome called Hurler–Scheie syndrome [40]. When taken together, our evidence shows the possibility of (1) taking newly found mutated genes from newborn screening and then (2) quantifying enzymatic activity combined with direct observation of phenotypic defect in zebrafish embryos, thereby (3) providing a new platform for preliminary assessment of such mutant genes as closely correlated with human MPS I or not.

### 4.4. Accumulated HS, but Not DS, Was Detected in idua-Knockdown Zebrafish Embryos, Resulting in the Occurrence of Edematous Tissues

We used LC-MS/MS to detect whether the metabolites DS and HS were accumulated in the *idua*-knockdown zebrafish embryos in which Idua enzymatic activity was reduced. Results showed that HS concentration was increased in z-*idua*-MO-injected embryos at 120 hpf. Unexpectedly, DS concentration was not significantly increased. Using reverse phase ion-pair HPLC, Habicher et al. [43] detected the quantities of HS and DS in zebrafish embryos at 30 hpf, 2 dpf, 3 dpf, and 4 dpf. They found that changes in the level of HS remained stable during the early developmental stages of zebrafish. In contrast, the sulfation of DS is a dynamic process dominated by variable proportions of 6-*O*- and 4-*O*-sulfated disaccharides, along with a minor attribution of 2-*O*-sulfated and disulfated disaccharides. Therefore, since HS is stable, it is easier to analyze any variation in total accumulation. However, dynamic changes in the expression level of DS complicate the identification of significant increases in total accumulation. Indeed, as noted above, using LC-MS/MS, we did not detect the accumulation of DS in the examined samples of 120 hpf zebrafish embryos in contrast to Filipek-Górniok et al. [44] who used alcian blue staining and demonstrated that DS was slightly accumulated in the cartilage and notochord of zebrafish embryos from 2 to 6 dpf, possibly explaining why we could observe the craniofacial cartilage defect and bending of the body axis in the z-*idua*-knockdown embryos. 

GAGs constitute the extracellular matrix (ECM) of cells. GAGs are large, charged molecules that allow the influx of water that causes swelling of the ECM [45]. When z-Idua enzymatic activity is reduced in zebrafish embryos, the accumulation of GAGs, such as HS, causes water to enter the embryos that they cannot expel, resulting in edematous tissues, such as the heart, eyes, and abdomen, common phenotypes found in z-*idua*-MO-injected embryos and an easily observed morphological defect, reflecting a key component of our dual assay strategy.

## 5. Conclusions

In this study, we found that z-Idua enzymatic activity of zebrafish *idua*-knockdown embryos was reduced, resulting in the accumulation of undegradable metabolite of heparan sulfate, as well as increased mortality and defective phenotypes similar to some symptoms of human MPS I. We suggest that the z-Idua enzyme activity assay combined with phenotypic observation of mutated-*idua*-injected zebrafish embryos could serve as an alternative platform for a preliminary assessment of mutated *IDUA* not yet characterized for their role in MPS I.

## Figures and Tables

**Table 1:** The primers used for site-directed mutagenesis of zebrafish *idua*.

Mutants	Forward Primers (5′-3′)	Reverse Primers (5′-3′)
z-*idua*-L346R	CTACACTCTGCGGAGCAATGATAACG	CGTTATCATTGCTCC-GCAGAGTGTAG
z-*idua*-T364M	CCAGCGCATGCTCACCGC	GCGGTGAGCATGCGC-TGG
z-*idua*-E398-deletion	CACTGTTAGGCACTC-AGGTGCAG	CTGCACCTGAGTGCC-TAACAGTG
z-*idua*-E540-frameshift	GCTCAGTCTGGGGGA-AATGCCC	GGGCATTTCCCCCAG-ACTGAGC

## Data Availability

Not applicable.

## Supplementary Materials

The following supporting information can be downloaded at: https://www.mdpi.com/article/10.3390/jpm12081199/s1, Figure S1: Sequence alignment and conservation between human and zebrafish IDUA proteins.

## Abbreviations

CNS	central nervous system
MPS	mucopolysaccharidosis
LSD	lysosomal storage disorder
IDUA	α-l-Iduronidase
hpf	hours post-fertilization
dpf	days post-fertilization
PFA	paraformaldehyde
GAGs	glycosaminoglycans
HS	heparan sulfate
DS	dermatan sulfate
MPS I-H	Hurler syndrome
MPS I-S	Scheie syndrome
MPS I-HS	Hurler–Scheie syndrome
HSCT	hematopoietic stem cell transplantation
ERT	enzyme replacement therapy
IDS	*iduronate 2-sulfatase*
MO	morpholino oligonucleotides
