# Supplementary material for: Quantification of Idua Enzymatic Activity Combined with Observation of Phenotypic Change in Zebrafish Embryos Provide a Preliminary Assessment of Mutated idua Correlated with Mucopolysaccharidosis Type I
Source: J Pers Med. 2022 Jul 23;12(8):1199. doi: 10.3390/jpm12081199 (PMC9332586; doi:10.3390/jpm12081199)
Supplement: Supplementary file 1 [file jpm-12-01199-s001.zip › jpm-1801243-supplementary.pdf]

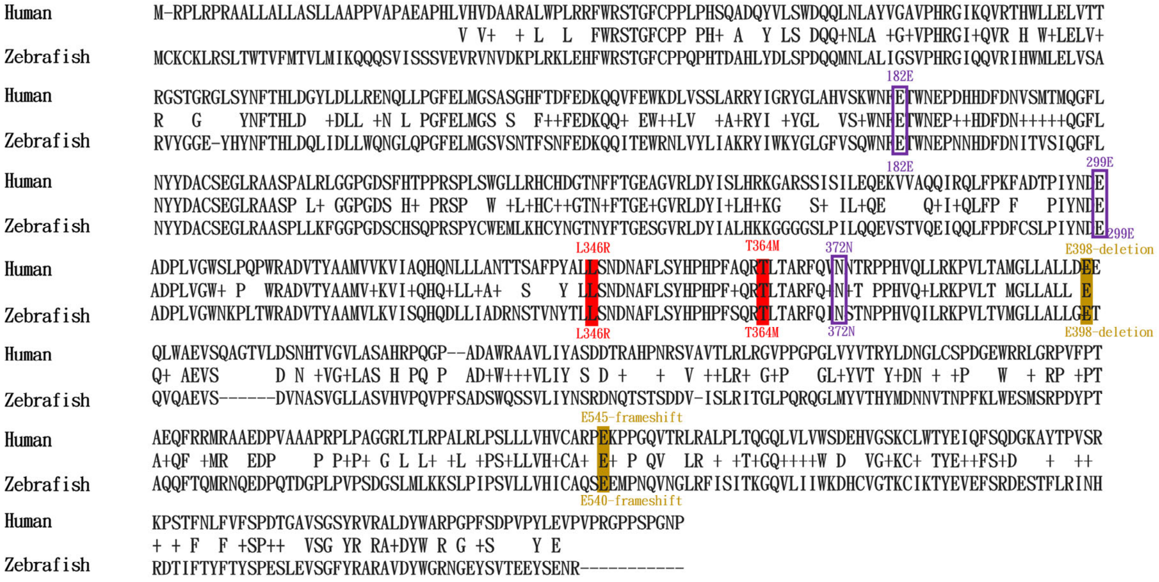

enzyme catalytic sites: Glu 182 and Glu 299; N-glycosylation site: Asn 372;
MPS I patients mutation sites: L346R, T364M, E398-del and E545-fra.

Figure S1. Sequence alignment and conservation between human and zebrafish IDUA proteins.
